# Exploring Coaches’ Strategies for Enhancing Athlete Happiness: A Q-Method Study of Subjective Psychosocial Perspectives

**DOI:** 10.3390/healthcare14040419

**Published:** 2026-02-07

**Authors:** Yavuz Öntürk, Vlad Adrian Geantă, Ahmet Yavuz Karafil, Esin Yilmaz, Vasile Emil Ursu, Borko Katanić

**Affiliations:** 1 Department of Sports Management, Faculty of Sports Sciences, Yalova University, Yalova 77100, Turkey; yavuzonturk@hotmail.com; 2 Department of Physical Education and Sport, Faculty of Physical Education and Sport, Aurel Vlaicu University of Arad, 310330 Arad, Romania; 3 Department of Sport Management, Faculty of Sports Sciences, Burdur Mehmet Akif Ersoy University, Burdur 15030, Turkey; aykarafil@mehmetakif.edu.tr (A.Y.K.); esinyilmaz@mehmetakif.edu.tr (E.Y.); 4 Department of Physical Education and Sport, Faculty of Law and Social Sciences, University “1 Decembrie 1918” of Alba Iulia, 510009 Alba Iulia, Romania; 5 Montenegrin Sports Academy, 81000 Podgorica, Montenegro; borkokatanic@gmail.com

**Keywords:** happiness, sport psychology, coaching strategies, Q methodology, psychological well-being, self-determination theory

## Abstract

**Highlights:**

**What are the main findings?**

- Coaches adopt two distinct psychosocial orientations to enhance athlete happiness: a group-based approach focusing on team cohesion and collective support and an individual-centered approach emphasizing empathy, personal recognition, and emotional sensitivity.
- Q methodology revealed that these orientations coexist within the coaching population, highlighting nuanced perceptual patterns behind strategies aimed at promoting athlete well-being.

**What are the implications of the main findings?**

- Effective coaching requires balancing team dynamics and individualized support, suggesting that training programs should incorporate both collective and personal psychosocial strategies.
- Integrating psychological well-being as a core component of coaching education and organizational policy can enhance athletes’ emotional development and long-term performance sustainability.

**Abstract:**

**Background/Objectives**: Coaches substantially influence athletes’ psychological well-being, yet the specific strategies they use to enhance happiness remain insufficiently understood. Given the established contribution of happiness to motivation, resilience, and long-term sport engagement, identifying these strategies and the perceptual patterns underlying them is essential. This study examined coaches’ subjective viewpoints regarding happiness-oriented strategies and identified the psychosocial orientations that structure these perspectives. **Methods**: Q methodology was applied using a 30-item Q set developed from interviews and expert review. Thirty professional coaches (≥5 years of experience) ranked the items according to perceived importance. By-person factor analysis and z-score interpretation were used to derive shared viewpoints. **Results**: Two coherent factors emerged. Factor 1 (59% variance) reflected a group-oriented psychosocial support approach, emphasizing team cohesion, positive feedback, social support, and mental resilience. Factor 2 (9% variance) represented an individual-centered, empathy-driven orientation, characterized by value affirmation, personalized communication, and emotional sensitivity. Distinct z-score patterns underscored clear contrasts between collective and individualized strategies. **Conclusions**: Coaches promote athlete happiness through two complementary orientations: collective psychosocial support and individualized psychological sensitivity. These findings extend self-determination theory and positive psychology by demonstrating how relatedness, competence, and individualized care are operationalized within coaching practice. The results offer practical guidance for integrating well-being into coach education and organizational policies.

## 1. Introduction

Happiness is increasingly recognized as a fundamental aspect of individual well-being and has been extensively studied across psychology, sociology, economics, and sport sciences [1,2]. It encompasses both life satisfaction and the experience of positive emotions and is shaped by factors such as personal growth, achievement, and social relationships [3,4,5]. Recent conceptualizations further describe happiness as a multidimensional and dynamic construct that integrates emotional experiences, psychological functioning, and social connectedness, emphasizing sustainability rather than a transient positive effect [6,7]. Within sport contexts, athletes’ happiness is not only a key indicator of psychological well-being but also influences motivation, performance, and long-term engagement in training and competition [8,9,10].

Among the primary determinants of athletes’ happiness are coaches’ behaviors, leadership styles, and psychosocial support strategies [11,12,13]. Coaches’ ability to satisfy athletes’ psychological needs has been shown to significantly enhance motivation and emotional well-being [14]. Self-determination theory (SDT) provides a robust theoretical framework for understanding these processes, positing that the satisfaction of three basic psychological needs—autonomy, competence, and relatedness—is essential for fostering both well-being and motivation [15].

Although happiness may be experienced as a transient emotional state, in sport contexts it is increasingly conceptualized as a broader indicator of psychological well-being that is closely linked to motivational quality and achievement-related satisfaction [16,17]. From this perspective, happiness reflects not only momentary positive affect but also athletes’ longer-term sense of meaning, competence, and engagement in their sporting activities.

Self-determination theory is thus especially relevant, as it explains how meeting basic psychological needs promotes more stable and long-lasting forms of well-being and motivation than short-term emotional fluctuations. Autonomy refers to athletes’ capacity to make decisions and exert control over training, which enhances their sense of agency and happiness [18]. Competence involves opportunities for skill development and recognition of achievements, closely linked to coaches’ feedback and encouragement [8]. Relatedness reflects athletes’ need for meaningful connections and a supportive social environment, which can be strengthened by cohesive team dynamics and empathetic coaching practices [19]. In the present study, happiness is operationalized as a subjective experiential outcome reflecting athletes’ positive emotional engagement, perceived meaning, and satisfaction within the sport context. Although closely related to well-being and motivation, happiness is examined separately because it captures athletes’ lived and momentary evaluation of their sport participation, rather than broader psychological functioning or motivational regulation processes [1,2]. This distinction aligns with conceptual models that differentiate experiential happiness from global well-being and adaptive capacities [3,5].

Despite this theoretical foundation, most empirical research has used psychometric instruments or investigated general motivational climates rather than the specific tactics coaches use to improve athletes’ satisfaction [8,20,21,22]. Research explicitly investigating how coaches operationalize happiness-focused strategies and the subjective patterns that shape these approaches is notably limited [22]. Furthermore, the long-term influence of such tactics on psychological resilience and sustained well-being in athletes has yet to be investigated.

Recent sport psychology research has emphasized athlete well-being as a multifaceted construct influenced by coaching behaviors, leadership styles, and psychosocial climates, particularly in high-performance sport settings [9,13]. However, less is known about how coaches themselves conceptualize and prioritize strategies aimed at fostering athletes’ happiness, creating a notable gap in understanding the subjective mechanisms behind happiness-oriented coaching practices [7,22]. While most studies have relied on questionnaire-based and variable-centered approaches, these methods are limited in capturing the nuanced mental models and meaning-making processes of coaches [23].

To address this gap, the present study employed Q methodology, a mixed-methods approach that systematically identifies shared subjective perspectives and underlying psychosocial orientations [24,25,26,27]. By applying this approach, the study explores how coaches organize and prioritize strategies to enhance athlete happiness, providing insight into distinct psychosocial orientations that are often overlooked in conventional research.

Accordingly, this study aimed to examine coaches’ subjective viewpoints on happiness-oriented strategies and to identify the psychosocial orientations that structure these perspectives, thereby contributing to both theoretical understanding and practical guidance in sport psychology and coaching practice.

## 2. Materials and Methods

### 2.1. Research Design

This study employed a descriptive and exploratory design to identify strategies adopted by coaches to enhance athletes’ happiness and examine the perceptual patterns underlying these strategies. The descriptive component allowed systematic characterization of psychosocial support strategies without manipulating variables, while the exploratory aspect enabled investigation of underexamined subjective viewpoints and identification of distinct patterns in coaching behavior. Data collection occurred during the same competitive season to ensure consistency.

Q methodology, a mixed-methods approach integrating qualitative and quantitative techniques [24], was employed to systematically analyze participants’ subjective perspectives. This approach combines a structured ranking procedure with interpretive analysis, enabling the identification of latent perceptual patterns and typologies often overlooked by traditional survey methods [21,25,26,27,28,29]. To enhance rigor and reduce potential bias, triangulation was applied by integrating semi-structured interviews, literature-derived Q statements, expert validation, and by-person factor analysis, ensuring both trustworthiness and systematic examination of subjectivity.

Preliminary conceptual work, including literature review and development of the Q statement concourse, was conducted prior to ethics approval and did not involve participant recruitment, data collection, or interaction with human subjects.

### 2.2. Participants

The study sample consisted of 30 professional coaches actively working across various sports disciplines in different regions of Turkey, each with a minimum of five years of coaching experience. The sample included 15 males and 15 females, aged 30–50 years (M = 39.8 ± 5.6 years). In terms of coaching context, 15 participants coached team sports and 15 coached individual sports. This demographic information was collected to contextualize potential variations in coaching perspectives.

Participants were recruited using purposive sampling, a strategy designed to maximize the diversity of subjective viewpoints [30,31,32]. This approach aligns with a core assumption of Q methodology, which emphasizes the identification of meaning-based factor structures rather than statistical generalization [26,32].

In Q methodology research, sample sizes typically range from 12 to 60 participants, depending on the study objectives and the complexity of the phenomenon under investigation [26]. Within this framework, the inclusion of 30 coaches was considered sufficient to capture a broad spectrum of perspectives and to support robust factor extraction and interpretation of perceptual patterns in coaching strategies.

All procedures involving human participants were conducted in accordance with the Declaration of Helsinki, and the study received ethical approval from the Yalova University Social and Human Sciences Research Ethics Committee (Protocol No. 2025/48, 28 January 2025). Informed consent was obtained from all participants prior to data collection.

### 2.3. Data Collection Instrument

A demographic information form was administered to collect basic descriptive data on participants, including age, sport discipline, coaching experience, team level, and institutional affiliation. This contextual information facilitated the interpretation of variations in coaches’ perspectives and ensured that differences in background could be considered when analyzing subjective responses.

#### 2.3.1. Semi-Structured Interview Form

To gain deeper insights into strategies coaches employ to enhance athletes’ happiness, a semi-structured interview form was developed. The questions were informed by key literature in sport psychology and coaching [11,20,33] and refined through feedback from three field experts. The interview protocol addressed core themes, including psychological need support, team relationships, individual recognition, and empathetic approaches [34], allowing participants to articulate the rationale underlying their coaching decisions and to elaborate on contextual nuances.

#### 2.3.2. Q Statement Set (Q Set)

The Q set comprised 30 statements generated through a multi-step process involving literature review [2], thematic analysis of semi-structured interviews, and expert consultation. Statements were organized into six thematic domains representing key psychosocial support strategies: positive feedback, mental resilience, social support, individual recognition, empathetic understanding, and positive team relationships. To systematically capture diverse viewpoints, items were formatted as bipolar pairs, reflecting meaningful contrasts in coaching behaviors [35]. The development of the Q statement set was informed by both the literature review and the semi-structured interviews. The literature review provided a conceptual foundation by identifying key themes related to happiness, well-being, motivation, and coaching practices in sport contexts. Semi-structured interviews with coaches were then used to capture context-specific expressions and practice-oriented perspectives. Statements derived from these sources were integrated, refined, and reviewed by subject-matter experts to ensure clarity, relevance, and representativeness. This combined approach ensured that the final Q set reflected both theoretical grounding and lived coaching experience.

#### 2.3.3. Q Sorting Procedure

Participants were instructed to rank the 30 statements using a fixed quasi-normal distribution ranging from −5 (strongly disagree) to +5 (strongly agree) [26]. This forced-ranking method requires the evaluation of statements relative to one another in terms of perceived importance. Prior to the task, participants received detailed instructions and provided written informed consent. As the study involved no interventions or sensitive data collection, and all data were collected anonymously, it was classified as minimal-risk behavioral research under institutional guidelines.

### 2.4. Data Analysis

Q-sort data were analyzed using PQMethod software (version 2.35; developed by Peter Schmolck, Freie Universität Berlin, Berlin, Germany). Principal component analysis (PCA) was conducted to identify underlying factor structures, followed by Varimax rotation to enhance interpretability [34,35,36]. Participants with factor loadings ≥ 0.47 were considered to have significant loadings [37]. Factor validity was assessed using the eigenvalue ≥ 1.0 criterion. Both high and low z-score items, as well as distinguishing statements, were incorporated into factor interpretation to provide a comprehensive understanding of perceptual patterns.

### 2.5. Validity and Reliability

Content validity of the Q statement set was ensured through review by three academic experts specializing in sport psychology and coaching. Face validity was assessed for clarity, comprehensibility, and cultural appropriateness [38]. Construct validity was supported via expert consensus, emphasizing inclusivity and representativeness [39]. Reliability of the derived factor structures was evaluated using composite reliability coefficients, with both factors exceeding 0.90, indicating a high level of internal consistency and robustness [37].

## 3. Results

### 3.1. Thematic Distribution of Coaching Strategies

Table 1 presents the coaching strategies aimed at enhancing athlete happiness, organized thematically as bipolar pairs reflecting positive and negative perspectives. This design, consistent with Q methodology principles, facilitates the systematic classification of subjective viewpoints.

Thematic recurrences were particularly notable in domains such as positive feedback, mental resilience, and social support, highlighting coaches’ attention to key psychosocial factors.

**Figure 1:**
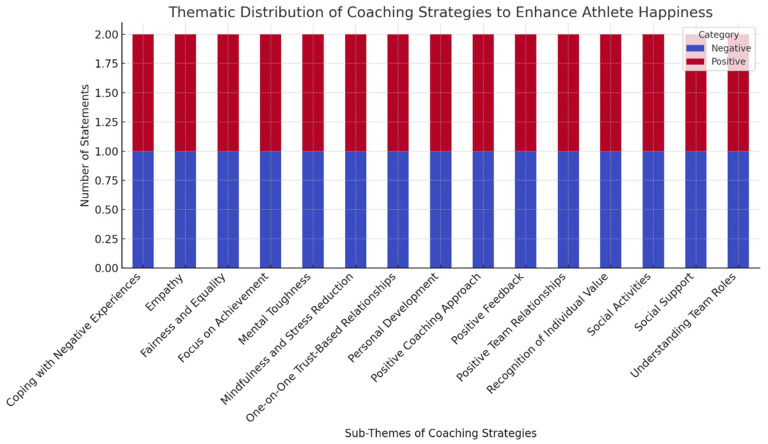
Analytical stages of the Q methodological procedure.

Figure 1 illustrates the main analytical stages of the Q methodological procedure, serving as a visual overview of the factor extraction process rather than a presentation of empirical results.

### 3.2. Factor Analysis

Principal component analysis (PCA) with varimax rotation identified two distinct factors. Factor 1 accounted for 59% of the variance and captured the dominant viewpoint, characterized by strong loadings on items such as Happy17 (0.913) and Happy12 (0.871), reflecting an emphasis on collective psychosocial support. Factor 2 accounted for 9% of the variance and represented a less prevalent but distinct perspective, defined by items such as Happy9 (0.567), Happy23 (−0.638), and Happy29 (0.618), emphasizing individual-centered, empathy-driven approaches. The factor structure and item loadings are reported in Table 2.

In line with the principles of Q methodology, the proportion of explained variance is not interpreted in terms of statistical dominance or generalizability. Instead, factors are evaluated based on their conceptual coherence and interpretive relevance. Accordingly, although Factor 2 accounts for a smaller proportion of variance, it represents a distinct and meaningful minority viewpoint that contributes to understanding alternative psychosocial orientations among coaches.

### 3.3. Z-Score Analysis

Analysis of Z-scores further distinguished the factors. Factor 1 participants strongly endorsed collective strategies, including enhancing mental resilience, fostering social support, and organizing team activities. Table 3 displays the highest and lowest Z-score items for Factor 1, demonstrating the prioritization of group-oriented psychosocial interventions.

Factor 2 participants emphasized individual-centered approaches, such as making athletes feel valued, adopting a positive attitude, and supporting empathy. The corresponding ranked Z-scores for Factor 2 are presented in Table 4.

**Figure 2:**
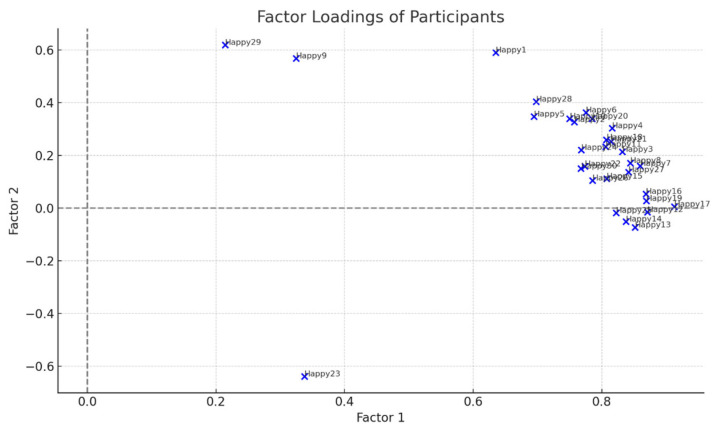
Participants’ factor loading chart.

**Figure 3:**
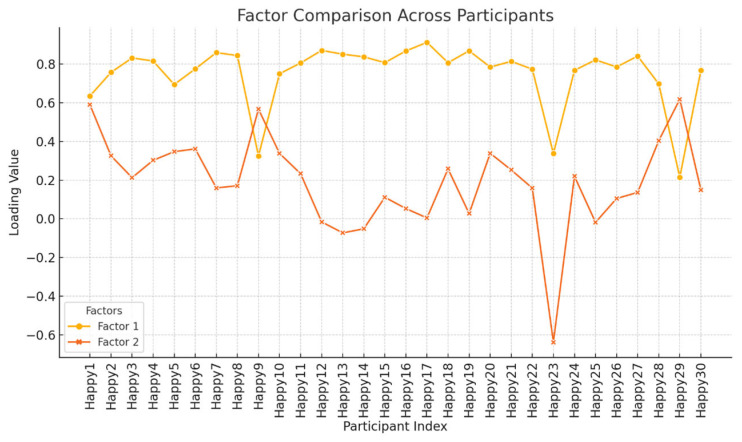
Factor comparison chart.

The distribution charts (Figure 2 and Figure 3) show participants’ degree of association with Factors 1 and 2. Most participants exhibited higher loadings on Factor 1, while Factor 2 captured a minority perspective, highlighting the differential emphasis on collective versus individual psychosocial strategies.

### 3.4. Distinguishing Statements

Statements exhibiting significant differences between factors were identified as distinguishing items, revealing core variations in coaching philosophy. Factor 1 strongly endorsed strategies enhancing group well-being, whereas Factor 2 emphasized individual-centered elements, including personalized attention, empathy, and positive communication. Distinguishing statements between factors are summarized in Table 5.

**Figure 4:**
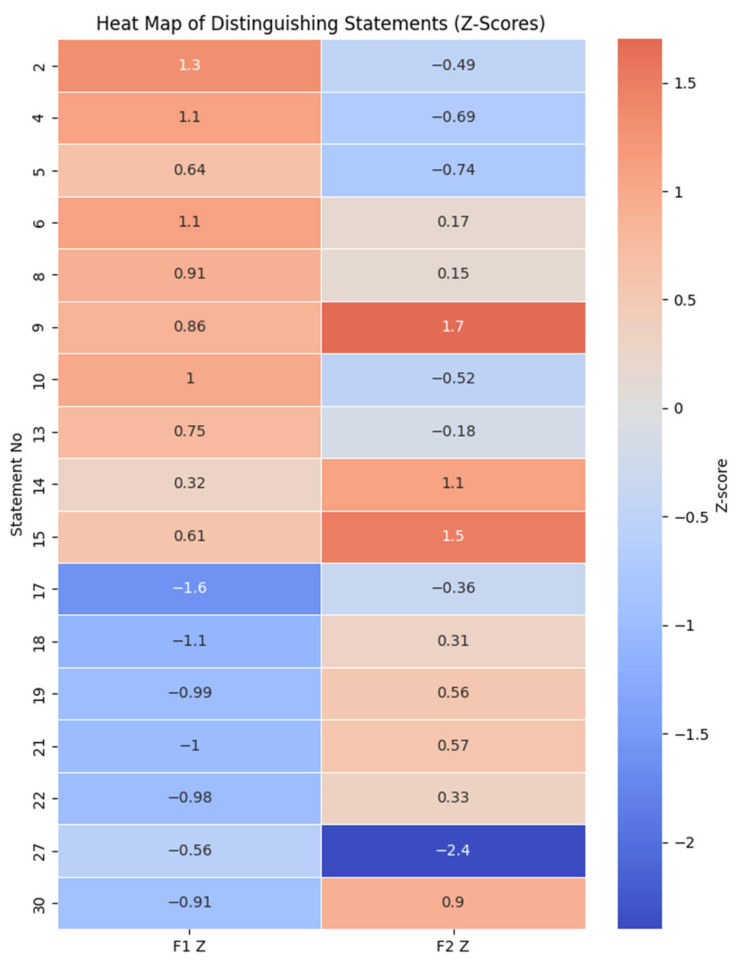
Heat map of distinguishing statements.

Figure 4 provides a visual summary of distinguishing and consensus statements; however, the substantive interpretation of these patterns is presented in the accompanying text.

## 4. Discussion

The findings of this study highlight two overarching psychosocial orientations that characterize coaches’ strategies to enhance athlete happiness: a group-oriented dimension grounded in collective support and solidarity and an individual-centered dimension shaped by empathy, personal recognition, and emotional sensitivity.

From a Q methodological perspective, the unequal proportion of explained variance across the two factors should not be interpreted in terms of statistical dominance or representativeness. Unlike variable-centered approaches, Q methodology is designed to identify shared patterns of subjectivity rather than to generalize findings to a population. Accordingly, minority factors are considered theoretically and interpretively meaningful, as they capture coherent alternative mental models. In this study, although Factor 2 explains a smaller proportion of variance, it represents a distinct and valuable individualized orientation toward athlete happiness that complements the dominant group-oriented perspective.

The first factor reflects coaching approaches that promote shared experiences, social cohesion, and resilience-building within the team environment. Coaches who strongly aligned with this factor emphasized structured social interactions, positive feedback, and mental resilience practices, elements that collectively strengthen athletes’ sense of belonging and contribute to a supportive motivational climate [7]. These strategies suggest that happiness is conceptualized as a collective outcome emerging from team cohesion, mutual support, and shared psychological resources.

The second factor reflects an individual-centered, empathy-driven coaching orientation in which athletes’ happiness is fostered through personalized attention, emotional sensitivity, and trust-based relationships, consistent with contemporary research emphasizing the central role of the coach–athlete relationship in psychological well-being [38,40]. This perspective suggests that, for some coaches, athlete happiness is not primarily understood as a collective phenomenon but rather as an outcome shaped by individualized relational dynamics and interpersonal understanding.

Interpreting these orientations through the lens of self-determination theory (SDT) [11,14] provides a deeper understanding of how coaching behaviors contribute to well-being. The subjective orientations identified in this study appear to be shaped by multiple interrelated factors, including coaches’ professional socialization, value orientations, and the contextual demands of competitive sport.

An exploratory, non-inferential examination suggested that coaches in team sports emphasized collective structure and group-oriented strategies, whereas coaches in individual sports favored individualized and autonomy-supportive approaches. Similarly, coaches in performance-driven environments prioritized discipline and resilience-building, while those with humanistic coaching philosophies highlighted empathy, personal recognition, and emotional sensitivity. These patterns reflect broader value-based and context-dependent coaching approaches described in contemporary sport psychology literature [7,21,33].

The group-centered strategies observed in Factor 1 align closely with the need for relatedness [19], as positive team relationships and shared psychosocial support help cultivate a sense of belonging. Elements of competence also emerge in practices such as reinforcing personal achievements and structured positive feedback, which align with athletes’ desire to feel effective and successful [32,41]. In contrast, Factor 2’s individual-centered approach resonates strongly with autonomy and emotional validation components of well-being [14,42,43], suggesting that empathy, recognition of individuality, and personalized communication facilitate the internalization of positive experiences and contribute to the development of self-worth.

The coexistence of these two factors indicates that coaching strategies aimed at enhancing happiness are not mutually exclusive; rather, they appear to operate along a continuum in which group cohesion and individual sensitivity reinforce one another. This interpretation aligns with previous research emphasizing the complementary role of psychological skills training and social support in promoting positive developmental outcomes [8,44]. Reinboth, Duda, and Ntoumanis [21] similarly found that coaches who foster mastery-oriented climates characterized by fairness, encouragement, and a developmental focus contribute to both the psychological and physical well-being of athletes. The present findings extend this literature by revealing the perceptual structures underlying these strategies, demonstrating that while many coaches embed happiness-enhancing practices within group contexts, others rely more heavily on dyadic and individualized interactions.

The identification of mindfulness practices, cognitive reframing strategies, and positive emotional communication as distinguishing elements across both factors provides further insight into the expanding influence of positive psychology within sport contexts. Seligman’s well-being theory [2] emphasizes the role of positive emotions, engagement, and supportive relationships in fostering human flourishing, and the present findings suggest that coaches may intuitively incorporate such principles even in the absence of formal psychological training. Empirical evidence has shown that mindfulness-based interventions can reduce stress and burnout among athletes [43], while practices such as positive reframing support adaptive coping and psychological resilience [45]. The prominence of these strategies within coaches’ subjective viewpoints underscores a growing recognition that athlete happiness, as a key component of psychological well-being, is integral to both performance and long-term athlete development. Mashhoot et al. [46] showed that mindfulness-based training improves athletes’ self-confidence and motivation through enhanced emotional regulation and attentional control. Consistent with the present findings, this indicates that coaches’ use of mindfulness and emotional support strategies fosters athlete happiness through adaptive psychological mechanisms rather than performance outcomes alone.

Despite this positive orientation, the persistence of performance-centric and discipline-focused beliefs among a subset of participants indicates that traditional coaching norms remain influential within sport culture. Such views echo concerns raised in previous research regarding the enduring tension between performance-driven expectations and holistic athlete development [21,33]. While these approaches may prioritize short-term performance outcomes, they risk neglecting the emotional and relational dimensions that support sustained motivation and long-term engagement. The present findings indicate that coaches endorsing these traditional perspectives constitute a minority viewpoint, suggesting a gradual shift toward more humanistic and psychosocially informed coaching philosophies.

Collectively, this study advances the sport psychology literature by elucidating not only the strategies coaches employ to enhance athlete happiness but also the perceptual frameworks that shape these practices. The application of Q methodology enabled a systematic examination of subjective viewpoints, uncovering nuances in how coaches conceptualize happiness-enhancing strategies that are often obscured by standardized quantitative approaches. This contribution extends existing research, which has predominantly focused on predefined constructs and outcome measures, by offering deeper insight into the subjective reasoning underlying coaching behaviors. It is also important to note that the present study examines coaches’ perceptions of strategies they believe promote athlete happiness, rather than athletes’ lived experiences or self-reported well-being. Accordingly, future research should directly examine athletes’ perspectives to further validate and extend these findings.

### Limitations

This study has several limitations. First, the purposive sample of 30 coaches from Turkey limits generalizability across other cultural or sporting contexts. Second, the study relied on self-reported perceptions rather than observational or performance-based data, which may introduce response bias. Third, the cross-sectional design prevents conclusions about the long-term impact of these coaching strategies on athlete well-being. Additionally, while Q methodology captures subjective viewpoints effectively, it does not quantify actual outcomes in athletes’ performance or mental health. Future research should address these limitations by employing longitudinal, multi-sport, and cross-cultural designs, as well as incorporating athlete-reported outcomes to triangulate the findings. A key limitation of this study is the absence of athletes’ perspectives. While the Q methodological approach provides valuable insight into how coaches conceptualize and prioritize happiness-oriented strategies, it does not allow conclusions to be drawn regarding athletes’ actual experiences of happiness or well-being. Future research should incorporate athletes’ voices, either through complementary Q studies or mixed-methods designs, to examine the extent to which these coaching orientations align with athletes’ perceptions and outcomes.

## 5. Conclusions

This study contributes to the sport psychology literature by clarifying how coaches conceptualize and prioritize happiness-oriented practices within their everyday work. Using Q methodology, the findings illustrate that coaches’ approaches to athlete happiness are structured around distinct but complementary psychosocial emphases, reflecting different ways of integrating well-being into coaching practice rather than a single dominant model.

From an applied perspective, the results underscore the relevance of addressing psychological well-being within coach education and organizational frameworks, alongside performance-related objectives. Rather than promoting a uniform set of strategies, the findings point to the value of fostering coaches’ reflective capacity to adapt their relational and psychosocial approaches to contextual and individual demands.

Importantly, these conclusions should be interpreted as exploratory rather than prescriptive, consistent with the epistemological foundations of Q methodology. The present study captures coaches’ subjective viewpoints rather than athletes’ lived experiences. Accordingly, future research should examine how these coaching orientations are perceived by athletes and how they relate to athletes’ happiness and well-being outcomes across diverse sporting, developmental, and cultural contexts.

## Figures and Tables

**Table 1:** Distribution of coaches’ happiness strategies by category and sub-themes.

Item No	Statement	Category	Sub-Theme
1	I enhance athletes’ self-confidence by frequently providing positive feedback.	Positive	Positive Feedback
16	Providing constant positive feedback is unnecessary; it does not contribute to extra motivation.	Negative	
2	I support athletes’ performance and happiness by increasing their mental resilience.	Positive	Mental Resilience
17	Mental resilience is not essential; physical training is sufficient.	Negative	
3	I conduct mindfulness exercises to reduce stress and increase happiness during competitions.	Positive	Mindfulness and Stress Reduction
18	Mindfulness exercises are unnecessary; athletes should focus directly on performance.	Negative	
4	I emphasize athletes’ personal achievements to strengthen their positive emotions.	Positive	Focus on Achievement
19	Emphasizing personal achievements is unnecessary; success is reflected in team performance.	Negative	
5	I reframe negative experiences to help athletes cope better with adversity.	Positive	Coping with Adversity
20	Reframing negative experiences is a waste of time; coping is a natural process.	Negative	
6	I promote athletes’ happiness by increasing social support within the team.	Positive	Social Support
21	Social support has little impact on happiness; individual success is more important.	Negative	
7	I strengthen team spirit by fostering positive relationships among athletes.	Positive	Positive Team Relationships
22	Fostering positive relationships is unnecessary; distance among athletes is healthier.	Negative	
8	I enhance athletes’ happiness by building trust-based one-on-one relationships.	Positive	Trust and One-on-One Relationships
23	Trust-based one-on-one relationships are not important; performance should be prioritized.	Negative	
9	I promote athletes’ happiness by making them feel individually valued.	Positive	Respect for Individual Value
24	Making athletes feel individually valued is unnecessary; only success matters.	Negative	
10	I organize camps, events, and non-competitive activities to enhance athletes’ happiness.	Positive	Social Activities
25	Organizing social events is a waste of time; training focus is more efficient.	Negative	
11	I support athletes not only in performance but also in personal development.	Positive	Personal Development
26	Supporting personal development is unnecessary; being success-oriented is enough.	Negative	
12	I enhance athletes’ sense of belonging and happiness by treating them fairly and equally.	Positive	Justice and Equality
27	It is unnecessary to treat athletes fairly and equally; some deserve more support.	Negative	
13	I help athletes understand and accept their roles within the team to enhance their happiness.	Positive	Role Understanding
28	Helping athletes understand roles is unnecessary; roles emerge naturally over time.	Negative	
14	I support athletes’ happiness by approaching them with empathy.	Positive	Empathy
29	Empathy is unnecessary; maintaining discipline is more important.	Negative	
15	I adopt a positive attitude to strengthen athletes’ emotional well-being and team relationships.	Positive	Positive Approach
30	Adopting a positive attitude is not important; direct warnings are more effective.	Negative	

**Table 2:** Factor loadings for principal components (varimax rotation).

QSORT	Factor 1 Loading	Mark 1	Factor 2 Loading	Mark 2
Happy1	0.635	x	0.590	x
Happy2	0.757	x	0.326	
Happy3	0.832	x	0.213	
Happy4	0.816	x	0.303	
Happy5	0.695	x	0.347	
Happy6	0.775	x	0.362	
Happy7	0.860	x	0.160	
Happy8	0.844	x	0.171	
Happy9	0.325		0.567	x
Happy10	0.750	x	0.338	
Happy11	0.806	x	0.233	
Happy12	0.871	x	−0.016	
Happy13	0.852	x	−0.073	
Happy14	0.838	x	−0.052	
Happy15	0.808	x	0.111	
Happy16	0.868	x	0.053	
Happy17	0.913	x	0.005	
Happy18	0.807	x	0.258	
Happy19	0.869	x	0.027	
Happy20	0.785	x	0.339	
Happy21	0.814	x	0.252	
Happy22	0.774	x	0.159	
Happy23	0.337		−0.638	x
Happy24	0.768	x	0.220	
Happy25	0.822	x	−0.019	
Happy26	0.785	x	0.105	
Happy27	0.842	x	0.136	
Happy28	0.698	x	0.404	
Happy29	0.214		0.618	x
Happy30	0.768	x	0.150	

Factor 1 explained 59% of the variance, whereas Factor 2 accounted for 9% of the variance. “x” indicates the factor on which the statement shows the highest factor loading.

**Table 3:** Factor 1: ranked items by Z-scores.

Item No	Statement	Z-Scores
1	I enhance athletes’ self-confidence by frequently providing positive feedback.	1.408
2	I support athletes’ performance and happiness by increasing their mental resilience.	1.320
3	I conduct mindfulness exercises to reduce stress and increase happiness during competitions.	1.184
4	I emphasize athletes’ personal achievements to strengthen their positive emotions.	1.134
6	I promote athletes’ happiness by increasing social support within the team.	1.122
7	I strengthen team spirit by fostering positive relationships among athletes.	1.027
12	I increase athletes’ sense of belonging and happiness by treating them fairly and equally.	1.014
10	I organize camps, events, and non-competitive activities to enhance athletes’ happiness.	1.012
21	Social support has little effect on happiness; individual success is more important.	−1.012
26	Supporting personal development is unnecessary; focusing on success is enough.	−1.017
23	Trust-based one-on-one relationships are not important; performance should be prioritized.	−1.018
16	Providing constant positive feedback is unnecessary; it doesn’t aid motivation.	−1.041
18	Mindfulness exercises are unnecessary; athletes should focus directly on performance.	−1.125
24	Making athletes feel individually valued is unnecessary; only success matters.	−1.236
17	Developing mental resilience is not crucial; physical training is sufficient.	−1.562

**Table 4:** Factor 2: ranked items by Z-scores.

Item No	Statement	Z-Scores
9	I promote athletes’ happiness by making them feel individually valued.	1.685
15	I adopt a positive attitude to strengthen athletes’ emotional well-being and relationships.	1.473
1	I enhance athletes’ self-confidence by frequently providing positive feedback.	1.389
3	I conduct mindfulness exercises to help athletes reduce stress and increase happiness.	1.266
7	I strengthen team spirit by fostering positive relationships among athletes.	1.099
14	I support athletes’ happiness by approaching them with empathy.	1.072
23	Building trust-based one-on-one relationships is not important; performance should be the focus.	−1.111
24	Making athletes feel individually valued is unnecessary; only success matters.	−1.112
16	Providing constant positive feedback is unnecessary; it does not contribute to extra motivation.	−1.480
27	It is unnecessary to treat athletes fairly and equally; some deserve more support.	−2.415

**Table 5:** Distinguishing statements by factor.

Item No	Statement	F1 Q-SV	F1 Z-Score	F2 Q-SV	F2 Z-Score
2	I support athletes’ performance and happiness by increasing their mental resilience.	4	1.32	−1	−0.49
4	I emphasize athletes’ personal achievements to strengthen their positive emotions.	3	1.13	−2	−0.69
6	I promote athletes’ happiness by increasing social support within the team.	3	1.12	0	0.17
10	I organize social activities to enhance athletes’ happiness.	2	1.01	−1	−0.52
8	I enhance athletes’ happiness by building trust-based one-on-one relationships.	2	0.91	0	0.15
9	I promote athletes’ happiness by making them feel individually valued.	1	0.86	5	1.69
13	I help athletes understand and accept their roles within the team to enhance their happiness.	1	0.75	0	−0.18
5	I reframe negative experiences to help athletes cope better with adversity.	1	0.64	−2	−0.74
15	I adopt a positive attitude to strengthen athletes’ emotional well-being and relationships.	0	0.61	4	1.47
14	I support athletes’ happiness by approaching them with empathy.	0	0.32	3	1.07
27	It is unnecessary to treat athletes fairly and equally; some deserve more support.	0	−0.56	−5	−2.41
30	Adopting a positive attitude is not important; direct warnings are more effective.	−1	−0.91	2	0.90
22	Fostering positive relationships is unnecessary; distance among athletes is healthier.	−2	−0.98	1	0.33
19	Emphasizing personal achievements is unnecessary; success is reflected in the team’s performance.	−2	−0.99	1	0.56
21	Social support has little impact on happiness; individual success is more important.	−2	−1.01	1	0.57
18	Mindfulness exercises are unnecessary; athletes should focus directly on performance.	−4	−1.12	1	0.31
17	Mental resilience is not essential; physical training is sufficient.	−5	−1.56	0	−0.36

## Data Availability

The raw data supporting the conclusions of this article will be made available by the authors upon request.

## Abbreviations

The following abbreviations are used in this manuscript:

SDT	Self-Determination Theory
PCA	Principal Component Analysis
